# The Role of DPO-1 and XE991-Sensitive Potassium Channels in Perivascular Adipose Tissue-Mediated Regulation of Vascular Tone

**DOI:** 10.3389/fphys.2016.00335

**Published:** 2016-08-04

**Authors:** Dmitry Tsvetkov, Jean-Yves Tano, Mario Kassmann, Ning Wang, Rudolf Schubert, Maik Gollasch

**Affiliations:** ^1^Experimental and Clinical Research Center, A Joint Cooperation between the Charité Medical Faculty and the Max Delbrück Center for Molecular Medicine in the Helmholtz Association of German Research CentresBerlin, Germany; ^2^Research Division Cardiovascular Physiology, Centre for Biomedicine and Medical Technology Mannheim, Medical Faculty Mannheim of the University HeidelbergMannheim, Germany; ^3^Medical Clinic for Nephrology and Internal Intensive Care, Charité University MedicineBerlin, Germany

**Keywords:** XE991, KCNQ channels, K_V_1.5 channels, adipocyte-derived relaxing factor (ADRF), perivascular adipose tissue (PVAT), BK channels

## Abstract

The anti-contractile effect of perivascular adipose tissue (PVAT) is an important mechanism in the modulation of vascular tone in peripheral arteries. Recent evidence has implicated the XE991-sensitive voltage-gated K_V_ (KCNQ) channels in the regulation of arterial tone by PVAT. However, until now the *in vivo* pharmacology of the involved vascular K_V_ channels with regard to XE991 remains undetermined, since XE991 effects may involve Ca^2+^ activated BK_Ca_ channels and/or voltage-dependent K_V_1.5 channels sensitive to diphenyl phosphine oxide-1 (DPO-1). In this study, we tested whether K_V_1.5 channels are involved in the control of mesenteric arterial tone and its regulation by PVAT. Our study was also aimed at extending our current knowledge on the *in situ* vascular pharmacology of DPO-1 and XE991 regarding K_V_1.5 and BK_Ca_ channels, in helping to identify the nature of K^+^ channels that could contribute to PVAT-mediated relaxation. XE991 at 30 μM reduced the anti-contractile response of PVAT, but had no effects on vasocontraction induced by phenylephrine (PE) in the absence of PVAT. Similar effects were observed for XE991 at 0.3 μM, which is known to almost completely inhibit mesenteric artery VSMC K_V_ currents. 30 μM XE991 did not affect BK_Ca_ currents in VSMCs. *Kcna5*^−/−^ arteries and wild-type arteries incubated with 1 μM DPO-1 showed normal vasocontractions in response to PE in the presence and absence of PVAT. K_V_ current density and inhibition by 30 μM XE991 were normal in mesenteric artery VSMCs isolated from *Kcna5*^−/−^ mice. We conclude that K_V_ channels are involved in the control of arterial vascular tone by PVAT. These channels are present in VSMCs and very potently inhibited by the KCNQ channel blocker XE991. BK_Ca_ channels and/or DPO-1 sensitive K_V_1.5 channels in VSMCs are not the downstream mediators of the XE991 effects on PVAT-dependent arterial vasorelaxation. Further studies will need to be undertaken to examine the role of other K_V_ channels in the phenomenon.

## Introduction

Over the past decade, various potassium (K^+^) channels have been implicated as important players in the regulation of arterial vascular tone and its control by perivascular adipose tissue (PVAT). Opening of vascular smooth muscle cell (VSMC) K^+^ channels causes K^+^ efflux and membrane hyperpolarization, which leads to reduced Ca^2+^ influx though L-type Ca_V_1.2 channels and consequently arterial relaxation (Nelson and Quayle, 1995). A variety of endogenous vasodilators, such as hypoxia, acidosis, as well as metabolites and autacoids (e.g., adenosine, prostacyclin) act as potent K^+^ channel openers to produce relaxation (Sobey, 2001; Tano and Gollasch, 2014). Noteworthy, many of these substances produce relaxation by opening maxi Ca^2+^ activated (BK_Ca_) K^+^ channels in VSMCs (Bentzen et al., 2014). Only very few substances have been reported to relax vessels by opening arterial smooth muscle voltage-gated K_V_ channels (Tanaka et al., 2006; Park et al., 2015). Among them adenosine and atrial natriuretic peptide (ANP) act *via* activation of the KCNQ (K_V_7) subfamily of K_V_ channels (Khanamiri et al., 2013; Stott et al., 2015).

Recent studies have demonstrated a paracrine role for PVAT to produce relaxation of arterial smooth muscle cells in a number of vascular beds (Lohn et al., 2002; Verlohren et al., 2004; Gao et al., 2005; Zavaritskaya et al., 2013). Certain adipokines, such as adiponectin (Weston et al., 2013), angiotensin-1 to 7 (Lee R. M. K. W. et al., 2011), methyl palmitate (Lee Y.-C. et al., 2011), and notably H_2_S (Schleifenbaum et al., 2010) were recently proposed as potential perivascular-derived relaxing factors (PVRFs), which could mediate the anti-contractile properties of PVAT. The paracrine effects of PVAT involve the opening of K^+^ channels, however, the identity of the K^+^ channel subtype(s) involved is still a matter of debate (Tano et al., 2014).

Voltage-gated K_V_ channels of the KCNQ (K_V_7) family have been proposed to play an important role in PVAT control of arterial tone. This conclusion is based on observations demonstrating that the anti-contractile effects of PVAT are inhibited by the pan KCNQ channel blocker XE991 at 30 μM or the pan K_V_ channel blocker 4-aminopyridine (2 mmol/L; Fésüs et al., 2007; Schleifenbaum et al., 2010; Lee Y.-C. et al., 2011; Zavaritskaya et al., 2013). XE991 is a widely used pan K_V_7 channel blocker, which inhibits K_V_7.1 homomeric or K_V_7.1/KCNE channels (IC_50_ of ~0.8 μM and 11.1 μM, respectively; Wang et al., 2000), KCNQ2/3 channels (EC_50_ ~1 μM; Wang et al., 1998), KCNQ4 (EC_50_ ~5.5 μM; Søgaard et al., 2001), and KCNQ5 (EC_50_ ~65 μM; Schroeder et al., 2000). Noteworthy, XE991 can also inhibit other K_V_ channels, such as ERG (K_V_11; EC_50_ ~110 μM) (Elmedyb et al., 2007) and K_V_1.2/1.5, K_V_2.1/K_V_9.3 channels (~30% inhibition at 10 μM) in heterologous expression systems (Zhong et al., 2010).

However, it is unknown whether XE991 is indeed specific for vascular K_V_ channels *in situ*, and does not inhibit native BK_Ca_ channels. This is particularly relevant since BK_Ca_ channels have been proposed to play a role in PVAT control of arterial tone in other studies (Lynch et al., 2013; Weston et al., 2013), although studies using BK_Ca_ deficient mice gave opposing results (Fésüs et al., 2007). A recent study showed that K_V_ channels in VSMCs of mouse mesenteric arteries are very sensitive to XE991 (EC_50_~60 nM), suggesting that these channels may contribute to PVAT control of arterial tone (Schleifenbaum et al., 2010, 2014). A very recent study suggested that diphenyl phosphine oxide-1 (DPO-1) sensitive K_V_1.5 channels could contribute to the K_V_ current in VSMC (Fancher et al., 2015).

Therefore, we tested whether K_V_1.5 channels are involved in the control of arterial tone and its regulation by PVAT or not. Our study is also aimed at extending our current knowledge on the *in situ* vascular pharmacology of DPO-1 and XE991 regarding K_V_1.5 and BK_Ca_ channels, in helping to identify the nature of K^+^ channels that could contribute to PVAT-mediated relaxation.

## Methods

### Mouse model

We used *Kcna5*^−/−^ mice as previously described (Pannasch et al., 2006). The mouse model was evaluated by RT-qPCR (Figure S1). Either litter- or age-matched (10–14 weeks old) male wild-type (129S6 background, previously known as 129SvEv-Ta) mice were used as controls. 250–300 g male Sprague Dawley rats were obtained from Charles River, Germany, Berlin. All experimental procedures were performed in accordance with the German legislation on protection of animals. Animal care followed American Physiological Society guidelines, and local authorities (Landesamt für Gesundheit und Soziales Berlin, *LAGeSo*) approved all protocols. Mice were housed in individually ventilated cages under standardized conditions with an artificial 12-h dark–light cycle with free access to water and food.

### Wire myography

First order mesenteric arteries were removed immediately after killing the mice or rats under inhalation anesthesia with isoflurane by cervical dislocation, quickly transferred to cold (4°C), oxygenated (95% O_2_/5% CO_2_) physiological salt solution (PSS) containing (in mmol/L) 119 NaCl, 4.7 KCl, 1.2 KH_2_PO_4_, 25 NaHCO_3_, 1.2 MgSO_4_, 11.1 glucose, 1.6 CaCl_2_), and dissected into 2 mm rings whereby perivascular fat and connective tissue were either intact [(+) PVAT or removed (−) PVAT] without damaging the adventitia. Each ring was positioned on two stainless steel wires (diameter 0.0394 mm) in a 5-ml organ bath of a Mulvany Small Vessel Myograph (DMT 610 M; Danish Myo Technology, Denmark). The organ bath was filled with PSS. The bath solution was continuously oxygenated with a gas mixture of 95% O_2_ and 5% CO_2_, and kept at 37°C (pH 7.4) (Verlohren et al., 2004; Fésüs et al., 2007). The mesenteric rings were placed under a tension equivalent to that generated at 0.9 times the diameter of the vessel at 100 mm Hg by stepwise distending the vessel using LabChart DMT Normalization module. This normalization procedure was performed to obtain the passive diameter of the vessel at 100 mm Hg (Fésüs et al., 2007). The software Chart5 (AD Instruments Ltd. Spechbach, Germany) was used for data acquisition and display. After 60 min equilibration arteries were pre-contracted either with isotonic external 60 mmol/L KCl until a stable resting tension was acquired. The composition of 60 mM KCl (in mmol/L) was 63.7 NaCl, 60 KCl, 1.2 KH_2_PO_4_, 25 NaHCO_3_, 1.2 Mg_2_SO_4_, 11.1 glucose, and 1.6 CaCl_2_. Drugs were added to the bath solution if not indicated otherwise. Tension is expressed as a percentage of the steady-state tension (100%) obtained with isotonic external 60 mM KCl.

### Isolation of arterial VSMCs

VSMCs from mesenteric arteries were isolated as described (Gollasch et al., 1998; Plüger et al., 2000). Briefly, the arteries were isolated and quickly transferred to cold (4°C) oxygenated (95% O_2_–5% CO_2_) PSS. The arteries were cleaned, cut into pieces, and placed into a Ca^2+^-free Hank's solution (in mmol/L): 55 NaCl, 80 sodium glutamate, 5.6 KCl, 2 MgCl_2_, 1 mg/ml bovine serum albumin (BSA, Sigma, Taufkirchen), 10 glucose, and 10 HEPES (pH 7.4 with NaOH) containing 0.5 mg/ml papain (Sigma) and 1.0 mg/ml DTT for 50 min at 37°C. The segments then were placed in Hank's solution containing 1 mg/ml collagenase (Sigma, type F and H, ratio 30 and 70%, respectively) and 0.1 mmol/L CaCl_2_ for 10 min at 37°C. Following several washes in Ca^2+^-free Hank's solution (containing 1 mg/ml BSA), single cells were dispersed from artery segments by gentle triturating. Cells were then stored in the same solution at 4°C.

### Electrophysiology

Voltage dependent potassium (K_V_) currents and BK_Ca_ currents were measured in the conventional whole-cell configuration of the patch-clamp technique at room temperature as previously described (Gollasch et al., 1996; Essin et al., 2007; Schleifenbaum et al., 2014). Patch pipettes (resistance 3–5 MΩ) for recording K_V_ currents were filled with a solution containing (in mmol/L): 130 KCl, 1 MgCl_2_, 3 Na_2_ATP, 0.1 Na_3_GTP, 10 HEPES, and 5 EGTA (pH 7.2; Yeung and Greenwood, 2005). Patch pipettes for recording BK_Ca_ currents contained (in mmol/L): 130 KCl, 1 MgCl_2_, 3 Na_2_ATP, 0.1 Na_3_GTP, 10 HEPES, 5 EGTA, and 4.3 CaCl_2_ (estimated [Ca^2+^] free, 10^−6^ mol/L; pH 7.2). The external bath solution contained (in mmol/L): 126 NaCl, 5 KCl, 1 MgCl_2_, 0.1 CaCl_2_, 11 glucose and 10 HEPES (pH 7.2; Yeung and Greenwood, 2005). Holding potential was −60 mV. Whole cell currents were recorded using an Axopatch 200B amplifier (Axon Instruments/Molecular Devices, Sunnyvale, CA, USA) or an EPC 7 amplifier (List, Darmstadt, Germany) and digitized at 5 kHz, using a Digidata 1440A digitizer (Axon CNS, Molecular Devices), and pClamp software versions 10.1 and 10.2 (Schleifenbaum et al., 2014).

### RT-qPCR

Total RNA was isolated from snap-frozen heart and aortae tissues with or without K_V_1.5 by using the RNeasy RNA isolation kit (Qiagen, Hamburg, Germany) according to the manufacturer's instruction. Isolated RNA concentration was measured and RNA quality was tested by NanoDrop-1000 spectrophotometer (PeqLab, Erlangen, Germany). For the synthesis of cDNA, equivalent amounts of RNA (2 μg) were used and processed by a high capacity cDNA reverse transcription kit (Life Technologies GmbH, Darmstadt, Germany). Quantitative analysis of target mRNA expression was performed with real-time PCR using the relative standard curve method (Markó et al., 2016). TaqMan or SYBR green analysis was conducted according to the manufacturer's instructions, using an Applied Biosystems 7500 Sequence Detector (Life Technologies Corporation, Carlsbad, CA, USA). The expression level of the target genes was normalized by the expression of 18S. Primers for were synthesized by Biotez (Berlin, Germany) and the sequences are as follows: K_V_1.5 Forward sequence: 5′-GCTACTTCGATCCCTTGAGAAAT-3′; Reverse sequence: AGTAGTACAAAATGCCATCGAAGCT, 18S Forward sequence: 5′-ACATCCAAGGAAGGCAGCAG-3′; Reverse sequence 5′-TTTTCGTCACTACCTCCCCG-3′.

### Materials

All salts and other chemicals were obtained from Sigma-Aldrich (Germany) or Merck (Germany). All drugs were freshly dissolved on the day of each experiment according to the material sheet. The following concentrations of drugs were used: phenylephrine (Sigma-Aldrich) ranged from 0.01 to 100 μmol/L, 5-HT from 0.01 to 10 μM, DPO-1 (Tocris) 1 and 10 μmol/L, 100 nmol/L iberiotoxin (Sigma Aldrich). XE991 (Tocris) was applied at concentrations between 0.3 and 30 μM.

### Statistics

Data represent mean ± SEM. EC_50_ values were calculated using a Hill equation: T = (B_0_ − Be)/(1 + ([D]/EC_50_)^n^) + Be, where T is the tension in response to the drug (D); Be is the maximum response induced by the drug; B_0_ is a constant; EC_50_ is the concentration of the drug that elicits a half-maximal response (Bychkov et al., 1998). Curve fittings were done by Prism 6 software using non-linear regression. Statistical significance was determined by two-way ANOVA or repeated-measures two-way ANOVA, followed by Bonferroni *post hoc* test, and using Prism 6 software. In case of unbalanced data, this software uses analysis of “unweighted means” to compare groups. Extra sum-of-squares *F*-test was performed for comparison of concentration-response curves and their 95% confidence intervals (CI). *P*-values < 0.05 were considered statistically significant. n represents the number of independent arteries tested or the number of cells measured. All rings were obtained from at least 3 different animals.

## Results

### Regulation of arterial tone by DPO-1 sensitive K_V_1.5 channels

**Figure 1:**
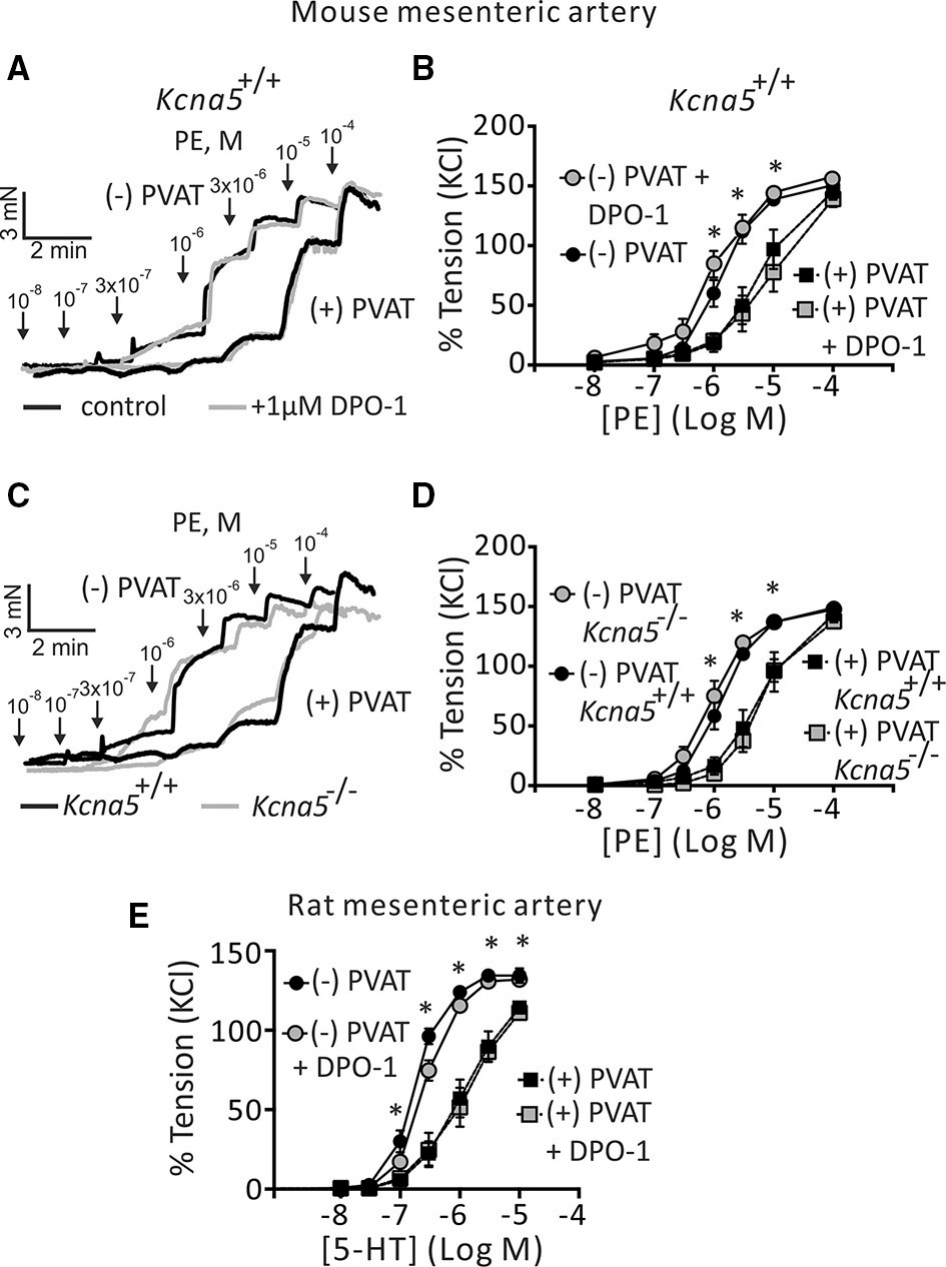
**Effects of pharmacological blockade and genetic deletion of K_**V**_1.5 channels on regulation of arterial tone by perivascular adipose tissue (PVAT), phenylephrine (PE), and serotonin (5-HT). (A)** Original traces showing the effects of 1 μM DPO-1 on PE-induced contractions in (−) PVAT and (+) PVAT mesenteric artery rings compared with control rings without DPO-1. **(B)** Concentration-response relationships for PE-induced contractions in *Kcna5*^+/+^ (+) PVAT (*n* = 8) or (−) PVAT (*n* = 9) mesenteric arteries in the absence of DPO-1 or in *Kcna5*^+/+^ (+) PVAT (*n* = 8) and (−) PVAT (*n* = 9) arteries after 30 min of pre-incubation with 1 μM DPO-1. ^*^p < 0.05, for (−) PVAT vs. (+) PVAT or (−) PVAT + DPO-1 vs. (+) PVAT + DPO-1; repeated-measures two-way ANOVA, followed by Bonferroni *post hoc* test. **(C)** Original traces showing the effects of genetic deletion of *Kcna5* on PE-induced contractions in (−) PVAT and (+) PVAT mesenteric artery rings. **(D)** PE-induced contractions of (+) PVAT and (−) PVAT artery rings isolated from *Kcna5*^+/+^ [(+) PVAT, *n* = 8; (−) PVAT, *n* = 9] and *Kcna5*^−/−^ [(+) PVAT, *n* = 12; (−) PVAT, *n* = 12] mice. ^*^p < 0.05, for (−) PVAT *Kcna5*^+/+^ vs. (+) PVAT *Kcna5*^+/+^ or (−) PVAT *Kcna5*^−/−^ vs. (+) PVAT *Kcna5*^−/−^. **(E)** Cumulative concentration-response relationships to 5-HT in (+) PVAT (*n* = 7) and (−) PVAT rat mesenteric arteries (*n* = 6) and the effects of 1 μM DPO-1 on (+) PVAT (*n* = 6) and (−) PVAT rat mesenteric arteries (*n* = 8). ^*^p < 0.05, for (−) PVAT vs. (+) PVAT or (−) PVAT + DPO-1 vs. (+) PVAT + DPO-1; repeated-measures two-way ANOVA, followed by Bonferroni *post hoc* test.

First, we examined the role of K_V_1.5 channels in the regulation of arterial tone by alpha1 adrenoceptor (alpha_1_-AR) stimulation. In this set of experiments, we used the K_V_1.5 channel blocker DPO-1 at concentrations assumed to be specific and potent for K_V_1.5 channel inhibition (Stump et al., 2005; Lagrutta et al., 2006; Regan et al., 2006). In the presence of 1 μM DPO-1, mesenteric artery rings without PVAT [(−) PVAT] displayed similar contractions in response to phenylephrine (PE) compared to non-treated (−) PVAT control rings (Figures 1A,B). The 95% CI for EC_50_ of control and DPO-1 treated rings were 1.21–1.79 μM and 0.78–1.40 μM, respectively. The anti-contractile effects of PVAT were also unchanged by 1 μM DPO-1: the 95% CI for EC_50_ of control (+) PVAT and 1 μM DPO-1 (+) PVAT treated rings were 3.99–8.14 μM and 4.99–10.05 μM, respectively. To further confirm the results obtained with the K_V_1.5 channel inhibitor, we performed similar experiments using mesenteric artery rings from *Kcna5*^−/−^ mice. PE induced vasocontractions in (−) PVAT *Kcna5*^−/−^ rings were not different from those observed in (−) PVAT *Kcna5*^+/+^ rings (Figures 1C,D). Similarly, we observed PE induced vasocontractions in (+) PVAT *Kcna5*^−/−^ rings, which were not different from those observed in (+) PVAT *Kcna5*^+/+^ rings (Figures 1C,D). The 95% CI for EC_50_ of (−) PVAT and (+) PVAT arteries isolated from *Kcna5*^−/−^ mice were 0.81–1.30 μM and 4.94–7.79 μM, respectively. Data are summarized in Table 1. Experiments on rat mesenteric arteries showed similar results: Cumulative dose-response curves in response to serotonin (5-HT) were similar in vessel rings in the absence or presence of DPO-1 (Figure 1E). Together, the results suggest that K_V_1.5 channels do not play a functionally relevant role in the control of arterial tone by PVAT, α1-AR and 5-HT agonists, in both mouse and rat mesenteric arteries.

**Table 1:** **EC_**50**_ and its confidence intervals**.

**Condition**	**Mouse background**	**Without PVAT**			**With PVAT**		
		**EC_50_ μM**	**95% confidence interval (CI)**	***n***	**EC_50_ μM**	**95% confidence intervals (CI)**	***n***
*Kcna5*^+/+^	129SVE-M	1.48	1.21–1.79	9	5.72	3.99–8.14	8
*Kcna5*^−/−^	129SVE-M	1.02	0.81–1.30	10	6.20	4.94–7.79	12
*Kcna5*^+/+^ +1 μM DPO-1	129SVE-M	1.04	0.78–1.40	11	7.24	4.99–10.05	11
Control	C57BL/6	0.70	0.57–0.87	17	4.94	4.18–5.84	21
0.3 μM XE991	C57BL/6	0.48	0.30–0.75	5	2.43	1.92–3.09	13
30 μM XE991	C57BL/6	0.38	0.24–0.65	5	1.64	1.32–2.02	12

*Data calculated from concentration-response curves to phenylephrine after normalization to maximum response from within each curve. For comparison of data and groups, see text and figures*.

### DPO-1 sensitive K_V_ channels distinct from K_V_1.5 may regulate arterial tone

**Figure 2:**
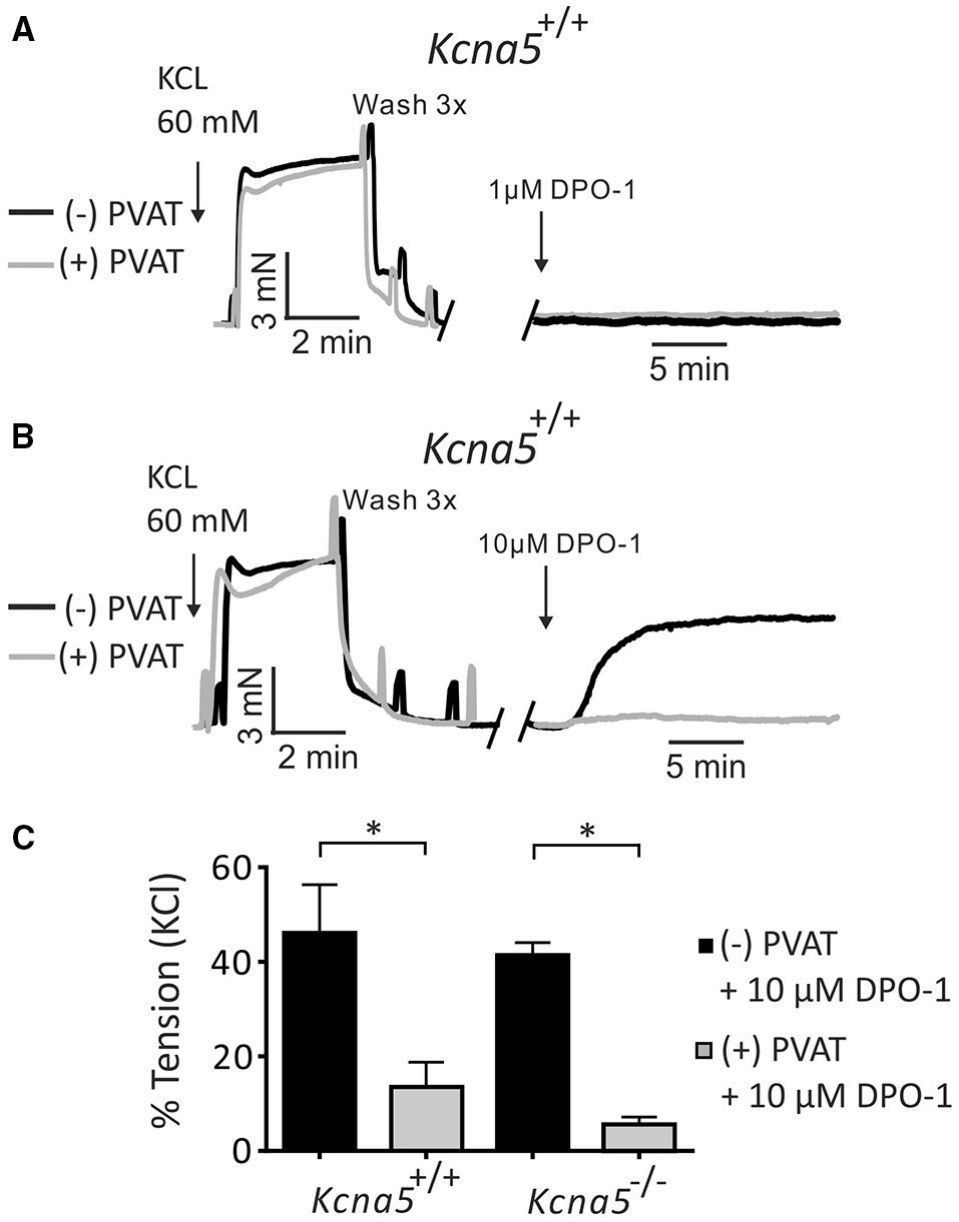
**Effects of 1 μM and 10 μM DPO-1 on basal arterial tone of mesenteric artery rings isolated from ***Kcna5***^**+/+**^ and ***Kcna5***^**−/−**^ mice**. Original traces showing contraction of mesenteric (−) and (+) PVAT rings induced by 60 mmol/L KCl and by 1 μM (**A**) and 10 μM DPO-1 (**B**). (**C**) Vessel tension induced by 10 μM DPO-1. Tension is expressed as a percentage of KCl contractions. *Kcna5*^+/+^ (−) PVAT, *n* = 9; (+) PVAT, *n* = 13. *Kcna5*^−/−^ (−) PVAT, *n* = 7; (+) PVAT, *n* = 9. ^*^p < 0.05, two-way ANOVA followed by Bonferroni *post hoc* test.

Next, we studied putative non-K_V_1.5 channel dependent effects by using higher concentrations of DPO-1. Figure 2A shows that 1 μM DPO-1 had no effects on basal tone of *Kcna5*^+/+^ mesenteric artery rings with and without PVAT. Surprisingly, application of 10 μM DPO-1 resulted in a stable contraction of *Kcna5*^+/+^ mesenteric arteries without but not with PVAT (Figure 2B). This effect remained stable over 30 min and was observed also on rings isolated from *Kcna5*^−/−^ mice (Figure 2C). Thus, unexpectedly, inhibition of DPO-1 sensitive K_V_ channels distinct from K_V_1.5 channels or other pathways could contribute to vascular tone in this preparation.

### Effects of XE991 on K_V_ currents, BK_Ca_ currents and the anti-contractile effects of PVAT

**Figure 3:**
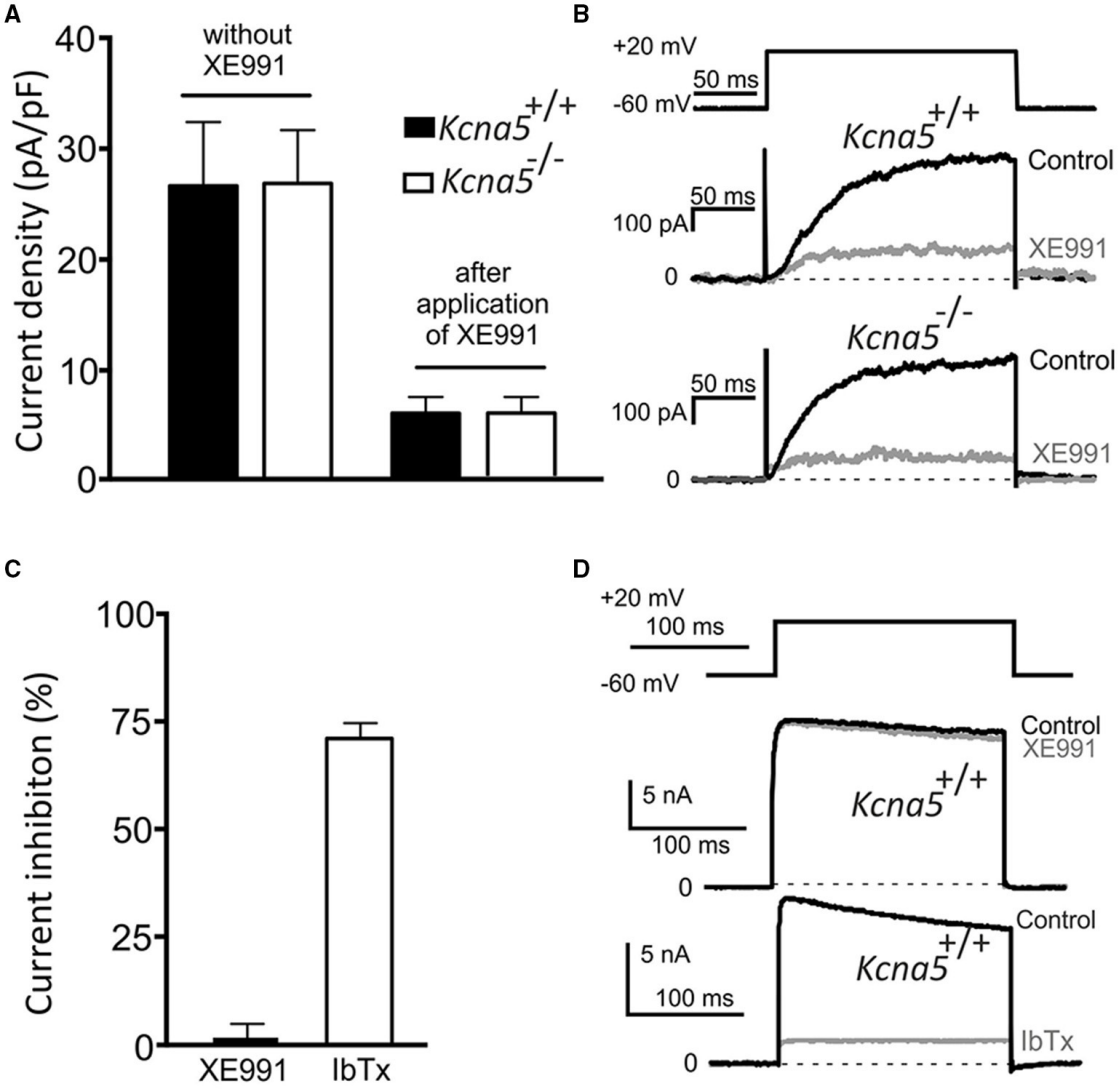
**Voltage dependent (K_**V**_) K^**+**^ currents and BK_**Ca**_ currents in freshly-isolated mesenteric artery vascular smooth muscle cells (VSMCs) from ***Kcna5***^**−/−**^ and ***Kcna5***^**+/+**^ mice**. K_V_ currents before (−XE991) and after (+XE991) application of 30 μM XE991. Current densities of peak K_V_ currents **(A)** (*n* = 6 *Kcna5*^−/−^ cells, *n* = 10 *Kcna5*^+/+^ cells, before and after application of XE991, respectively); original recordings **(B)**. **(C)** Relative inhibition of peak BK_Ca_ current by iberiotoxion (IbTx) at 100 nM (*n* = 5) and XE991 at 30 μM (*n* = 9) in *Kcna5*^+/+^ VSMCs, original recordings are represented in **(D)**.

K_V_ currents were recorded in mesenteric artery VSMCs freshly isolated from *Kcna5*^+/+^ and *Kcna5*^−/−^ mice. We did not observe any difference between K_V_ current densities in *Kcna5*^+/+^ and *Kcna5*^−/−^ VSMCs. Moreover, K_V_ current inhibition by 30 μM XE991 was not different between *Kcna5*^+/+^ and *Kcna5*^−/−^ VSMCs (Figures 3A,B). 30 μM XE991 did not affect basal tone of mesenteric arteries prepared with or without PVAT (Figure S2).

In order to better understand the effects of XE991, we tested its actions on BK_Ca_ currents, potential mediators of the PVAT effect. VSMC *Kcna5*^+/+^ BK_Ca_ currents were recorded in the absence and presence of 30 μM XE991. 100 nM iberiotoxin (a potent and highly selective BK_Ca_ channel inhibitor) was used as positive control. While 30 μM XE991 did not affect the BK_Ca_ current, iberiotoxin almost completely inhibited the BK_Ca_ current. These results are consistent with plasma membrane VSMC BK_Ca_ channel activity resistant to XE991 *in situ*, at concentrations up to 30 μM of XE991 (Figures 3C,D).

**Figure 4:**
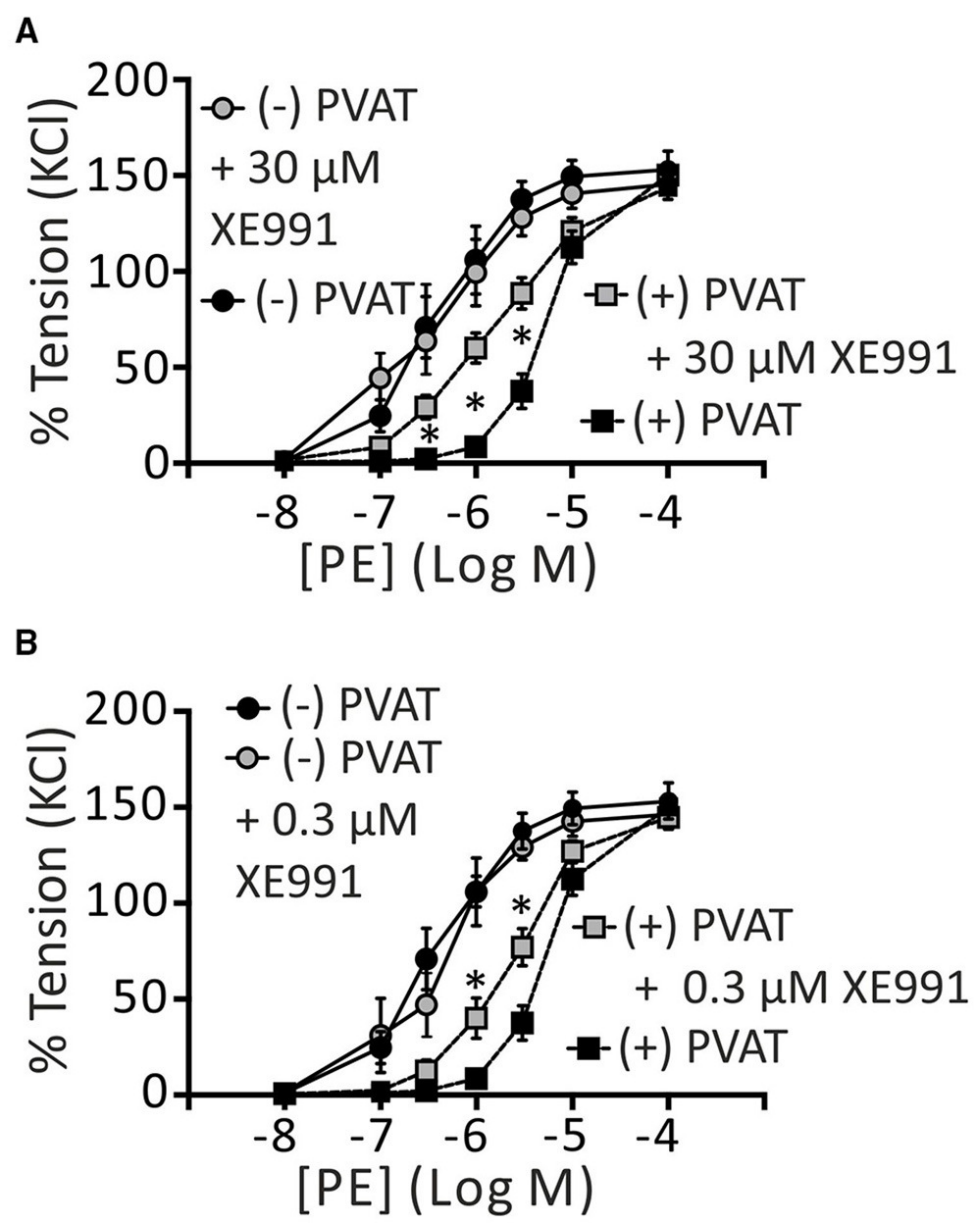
**Effects of XE991 on regulation of arterial tone by perivascular adipose tissue**. Cumulative concentration-response curves to PE in the presence of 30 μM XE991 **(A)** and 0.3 μM XE991 **(B)**, expressed as a percentage of KCl contractions. (−) PVAT, *n* = 17; (+) PVAT, *n* = 21. (+) PVAT (+) 30 μM XE991, *n* = 12; (+) PVAT + 0.3 μM XE991, *n* = 13; (−) PVAT (+) 30 μM XE991, *n* = 5; (−) PVAT + 0.3 μM XE991, *n* = 5. ^*^p < 0.05, repeated-measures two-way ANOVA, followed by Bonferroni *post hoc* test ^*^ in **(A)**, (+) PVAT vs. (+) PVAT + 30 μM XE991. ^*^In **(B)**, (+) PVAT vs. (+) PVAT + 0.3 μM XE991. Experiments were performed on mouse mesenteric arteries.

Additionally, we tested the effects of 30 μM and 0.3 μM XE991 on the paracrine effects of PVAT on arterial tone. We found that XE991 even at the low concentration abolished the anti-contractile effects of PVAT. Interestingly, application of 0.3 and 30 μM XE991 resulted in a similar reduction of the anti-contractile effects of PVAT (Figures 4A,B). The EC_50_ 95% CI values were 1.92–3.09 and 1.32–2.02 μM for (+) PVAT rings preincubated with 0.3 μM XE991 and 30 μM XE991, respectively; and 4.18–5.84 μM for (+) PVAT rings in the absence of XE991. (−) PVAT rings showed no difference, regardless of the absence or presence of 0.3 μM or 30 μM XE991. Data are presented in Table 1. Together, our data demonstrate that XE991 is a potent inhibitor of PVAT control of arterial tone at low concentrations similar to its potency of inhibiting K_V_ currents in VSMCs (Schleifenbaum et al., 2014). BK_Ca_ channels are however exempt from this inhibitory effect of XE991 (up to 30 μM).

## Discussion

Perivascular adipose tissue plays a potent anti-contractile role in the control of arterial tone along arterial segments of different vascular beds and species. The main findings of this study are threefold. First, XE991 inhibits the PVAT effect at nanomolar concentrations in mesenteric arteries of mice. Interestingly, similar concentrations were found in earlier studies to inhibit VSMC K_V_ currents (50% inhibition at 60 nM XE991) (Schleifenbaum et al., 2014). Second, *Kcna5*^−/−^ mice exhibited normal VSMC K_V_ currents and arterial contractions in the absence and presence of PVAT, whose effects were insensitive to DPO-1. Third, the KCNQ channel blocker XE991 does not affect plasma membrane VSMC BK_Ca_ channels at concentrations, which inhibit the anti-contractile effects of PVAT. Together, the results of our current study implicate KCNQ-type K_V_ channels in the XE991-mediated inhibition of the PVAT effects. Simultaneously, we exclude BK_Ca_ as well as K_V_1.5 channels as potential downstream candidates in this process.

### K_V_1.5 in regulation of arterial tone

In recent studies, K_V_1.5 channels have been shown to determine microvascular tone and the arteriolar response to vasoconstrictors in rat cerebral arteries (Chen et al., 2006; Fancher et al., 2015). Furthermore, K_V_1.5 channels in the heart are essential in coupling myocardial blood flow to cardiac metabolism (Ohanyan et al., 2015). Moreover, hypertension is associated with altered expression of vascular K_V_1.5 channels (Wang et al., 1997; Platoshyn et al., 2001; Cox and Rusch, 2002; Cox et al., 2008; Cidad et al., 2014). Therefore, K_V_1.5 channels may represent interesting putative targets of PVAT and that raised the question of their potential involvement in the regulation of arterial tone by phenylephrine in mouse mesenteric arteries. Our results suggest that K_V_1.5 channels are however not involved. In effect, the anti-contractile effects of PVAT were not different between *Kcna5*^−/−^ and *Kcna5*^+/+^ arteries. Additionally, the K_V_1.5 channel inhibitor DPO-1 at 1 μM displayed no effect on vasocontractions in the absence and presence of PVAT. Notably, the mechanism of DPO-1 action (“open channel” blocker) might be a potential confounding factor, since K_V_1.5 channels are activated at V_0.5_ of −14 mV (Grissmer et al., 1994). However, the genetic approach had the advantage to study vascular effects in the absence of K_V_1.5 channels avoiding possible confounders related to membrane potential-dependent drug mechanisms. Furthermore, we did not observe any significant differences in the K_V_ current density and inhibition by XE991 in *Kcna5*^−/−^ and *Kcna5*^+/+^ VSMCs. Therefore, we conclude that K_V_1.5 channels have no apparent role in PVAT-dependent relaxation and are not the XE991 sensitive channels that contribute to this process. This conclusion is in line with our previous results obtained on cloned and heterologously expressed K_V_1.5 alpha subunits in HEK293 cells (Schleifenbaum et al., 2014). In these experiments, 100 nM XE991 failed to block K_V_1.5 currents. We also observed similar responses of arterial rings without PVAT to PE and 5-HT, regardless of genetic deletion of K_V_1.5 alpha subunits or pharmacological blockade of K_V_1.5 channels by DPO-1. To our knowledge, this is the first study to firmly establish that K_V_1.5 channels are not involved in the regulation of arterial tone of systemic visceral arteries of mice and rats, at least in mesenteric arteries. Our conclusions substantiate the work of other groups, namely that patients with genetic mutations of *KCNA5* exhibit pulmonary arterial hypertension and arterial fibrillation but not systemic hypertension (Yang et al., 2009; Wipff et al., 2010; Machado et al., 2015). Together, our data questions the contribution of K_V_1.5 channels in a number of small resistance arteries to peripheral arterial resistance. The findings are however not generalizable to all vascular beds as K_V_1.5 channels were demonstrated to play important vasoregulatory functions in cerebral arteries and in *Gracilis* skeletal muscle arteries (Chen et al., 2006; Fancher et al., 2015).

### DPO-1 sensitive K^+^ channels and pathways distinct from K_V_1.5 involved in regulation of arterial tone

DPO-1 was described as a specific K_V_1.5 inhibitor at micromolar concentrations (Stump et al., 2005; Lagrutta et al., 2006; Regan et al., 2006). It exerts its inhibitory effects through binding with several key residues in the S5- pore loop-S6 domains, thus resulting in blockade of the open state of the K_V_1.5 channel (Karczewski et al., 2009; Du et al., 2010). Other studies have suggested a DPO-1 preference for vascular K_V_1.5 channels, though with limited selectivity (Fancher et al., 2015). In this study, DPO-1 (1–10 μM) inhibited the outward K^+^ current in arterial smooth muscle cells from wild-type (*Kcna5*^+/+^) mice and mice lacking the *Kcna5* gene; however, the inhibitory effect was much greater in cells from *Kcna5*^+/+^ mice (Fancher et al., 2015). Subsequently, our data in Figure 2 suggests that 10 μM DPO-1 induces contractions by inhibiting channels distinct from K_V_1.5 channels. Those channels appear to be important for the regulation of resting arterial tone. Interestingly, DPO-1 is able to block K_V_1.3 channel currents at EC_50_ of 3.1 μM in human T cells (Zhao et al., 2013). *Kcna3* mRNA expression is also observed in mouse mesenteric arteries (Fountain et al., 2004; Cidad et al., 2014). Although the ability of K_V_1.5 and K_V_1.3 to form heteromers (Kv1.5/Kv1.3) (Villalonga et al., 2007) impede the study of their specific roles in native tissues *in vivo*, it is intriguing to speculate that K_V_1.3 channels could represent a putative target of PVAT regulation of arterial tone. Future studies are necessary to clarify their role.

### Effects of XE991 on K_V_ channels, BK_Ca_ channels and paracrine PVAT effects

In the mouse mesenteric arteries, *Kcnq1, Kcnq4*, and *Kcnq5* expression was demonstrated at the mRNA level (Yeung et al., 2007), whereas mRNA expression of *Kcnq2, Kcnq3* was not detectable or only at borderline low levels (Yeung et al., 2007; Schleifenbaum et al., 2014). We previously suggested that KCNQ type K_V_ channels are key players in the paracrine role for periadventitial adipose tissue in the regulation of arterial tone (Schleifenbaum et al., 2010; Zavaritskaya et al., 2013); based on mRNA expression levels (see above), KCNQ1, KCNQ4, and/or KCNQ5 channels are likely candidates. This suggestion is also based on the ability of 30 μM XE991 (pan KCNQ blocker) and 2 mmol/L 4-aminopyridine (pan K_V_ blocker) to block the anti-contractile effects of PVAT. Interestingly, KCNQ channel openers normalized reduced anti-contractile effects of PVAT in a rat model of hypertension (Zavaritskaya et al., 2013), which suggests therapeutic perspectives of KCNQ targeting in cardiovascular disease. It is thus imperative to better understand the actions of these compounds on the vasculature. In order to obtain more information about the potency of XE991 inhibition on PVAT-mediated anti-contractility, we performed experiments with a 100x lower concentration of XE991. The data show that this considerably reduced concentration still exerted an inhibitory impact on PVAT regulation of arterial tone (Figures 4A,B), while basal tone was unaffected in rings (+) or (−) PVAT (Figure S2). The incomplete inhibition observed, however suggests the possible involvement of additional K^+^ channels in this process. Furthermore, our data demonstrates that VSMC plasma membrane BK_Ca_ channels are not involved in the effects of XE991 on PVAT regulation, since XE991 does not inhibit BK_Ca_ currents (this study). This is in line with our previous findings indicating that the paracrine effects of PVAT on arterial tone are normal in the presence of BK_Ca_ channel blockers or in arteries that lack BK_Ca_ beta1 channel subunits (Fésüs et al., 2007; Zavaritskaya et al., 2013).

Previous studies examined the ability of XE991 to inhibit heterologously expressed K_V_1.5 alpha subunits. We found that 100 nM and 30 μM XE991 was unable to block monotetrameric K_V_1.5 channels heterologously expressed in HEK293 or CHO cells (Zavaritskaya et al., 2013; Schleifenbaum et al., 2014). Interestingly, Zhong et al. found a small (~20% at (+) 5 mV) inhibiting effect for 10 μM XE991 on heterologously expressed heterotetrameric K_V_1.2/K_V_1.5 and K_V_2.1/K_V_9.3 channel subunits (Zhong et al., 2010). The block of K_V_1.2/K_V_1.5 channels was voltage dependent, and evident only at voltages positive to -15 mV. Our present study contributes to the debate about the importance of accessory subunits for determining the pharmacological properties of vascular K^+^ channels *in vivo*. Since regulatory K_V_beta1.3 subunits can decrease the sensitivity of K_V_1.5 channels to pharmacological inhibitors such as DPO-1 (Gonzalez et al., 2002; Arias et al., 2007; Du et al., 2010), one could argue that DPO-1 is not a reliable tool to study K_V_1.5 channels in native tissues. However, we believe that our pharmacological approach in combination with the *Kcna5*^−/−^ mouse model firmly demonstrates that the XE991 sensitive regulation of arterial tone by PVAT regulation does not involve native vascular K_V_1.5 channels.

## Conclusion

In conclusion, our results demonstrate that K_V_1.5 channels are not involved in the control of mesenteric arterial tone and its regulation by PVAT in mouse and rat mesenteric arteries. The nature of the 10 μM DPO-1 sensitive component is unclear, but is most likely related to non-specificity of this drug, for example in targeting vascular K_V_1.3 and/or KCNQ channels *in situ*. Importantly, the inhibitory effects of XE991 on PVAT vasorelaxation are rather related to inhibition of KCNQ-type K_V_ channels than BK_Ca_ channels. These data unequivocally substantiate the hypothesis of different targets of perivascular relaxing factor(s), which employ distinct mechanisms to mediate an anti-contractile effect. Further studies should focus on the enhancement of these relaxing factors, as these will be beneficial for patients with cardiovascular diseases.

## Author contributions

All authors planned and designed the experimental studies. DT and NW performed the wire myography experiments. MK and JT performed the electrophysiological experiments. DT and MG drafted the article, and all authors contributed to its completion.

## Funding

This study was supported by grants from the Deutsche Forschungsgemeinschaft (DFG) to MG and the Deutsche Akademische Austauschdienst (DAAD) to MG and DT. DT is recipient of ERA/EDTA and DAAD fellowships and JT is an Alexander von Humboldt fellow.

### Conflict of interest statement

The authors declare that the research was conducted in the absence of any commercial or financial relationships that could be construed as a potential conflict of interest.
