# Supplementary material for: The Role of DPO-1 and XE991-Sensitive Potassium Channels in Perivascular Adipose Tissue-Mediated Regulation of Vascular Tone
Source: Front Physiol. 2016 Aug 4;7:335. doi: 10.3389/fphys.2016.00335 (PMC4973012; doi:10.3389/fphys.2016.00335)
Supplement: Supplementary file 1 [file Presentation1.PDF]

Supplementary Material

The role of DPO-1 and XE991-sensitive potassium channels in perivascular adipose tissue-mediated regulation of vascular tone

Dmitry Tsvetkov[1#], Jean-Yves Tano,[1#] Mario Kaßmann,[1#] Ning Wang,[1] Rudolf Schubert[2], and Maik Gollasch[1, 3*]

[1]Experimental and Clinical Research Center (ECRC), a joint cooperation between the Charité Medical Faculty and the Max Delbrück Center for Molecular Medicine (MDC) in the Helmholtz Association the Helmholtz Association of German Research Centres, Lindenberger Weg 80, 13125 Berlin, Germany

[2]Centre for Biomedicine and Medical Technology Mannheim (CBTM), Research Division Cardiovascular Physiology, Medical Faculty Mannheim of the University Heidelberg, 68167 Mannheim, Germany

[3]Medical Clinic for Nephrology and Internal Intensive Care, Campus Virchow, Charité University Medicine, 13353 Berlin, Germany

#equal contribution

Maik Gollasch, MD PhD; maik.gollasch@charite.de

Keywords: XE991, KCNQ channels, K_v1.5 channels, adipocyte-derived relaxing factor (ADRF), perivascular adipose tissue (PVAT), BK channels

Figure Supplement 1. Validation of absence of mRNA of KCNA5 in *Kcna5*$^{-/-}$ mice (n=3 for heart isolated from *Kcna5*$^{+/+}$; n=5 for aorta isolated from *Kcna5*$^{+/+}$; n=5 for aorta isolated from *Kcna5*$^{-/-}$). Shown are medians and data points. *p<0.05, Mann-Whitney test.

Figure Supplement 2. Effects of 30 µM XE991 on basal arterial tone of mesenteric artery rings isolated from *Kcna5*$^{+/+}$ mice. **Panel A:** Original traces showing contractile responses of mesenteric (-) PVAT and (+) PVAT rings produced by 60 mmol/L KCl and by 30 µM XE991. **Panel B**: Tension produced by 30 µM XE991. Tension is expressed as a percentage of KCl contractions. *Kcna5*$^{+/+}$ (-) PVAT, n=7; (+) PVAT, n=7. The effects were not statistically significant.

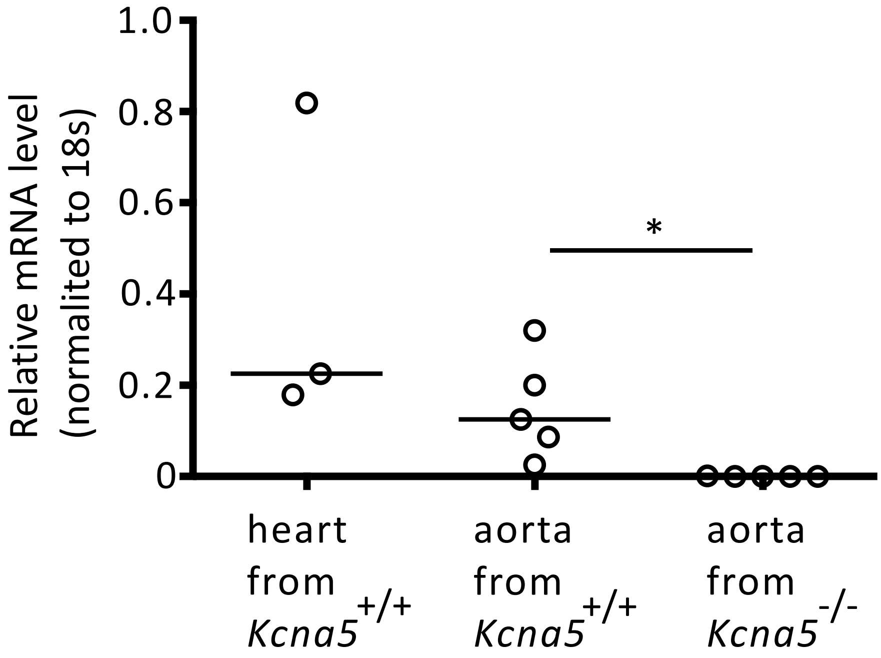
1.0
0.8
0.6
0.4
0.2
0
Relative mRNA level
(normalited to 18s)
*
heart from Kcna5+/+
aorta from Kcna5+/+
aorta from Kcna5-/-

Figure S1

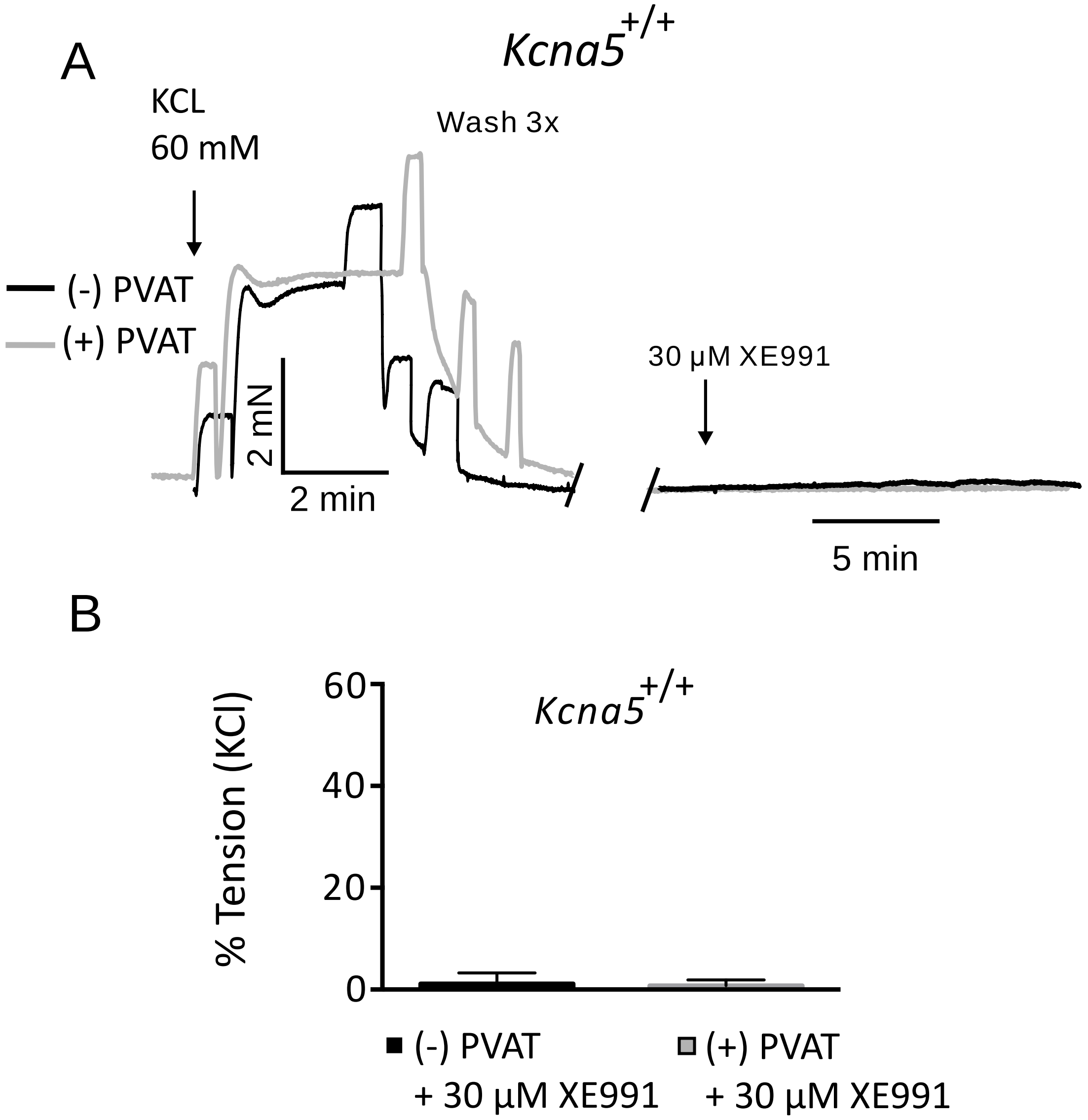
A
Kcna5+/+
KCL
60 mM
Wash 3x
(-) PVAT
(+) PVAT
2 mN
2 min
30 µM XE991
5 min
B
Kcna5+/+
% Tension (KCl)
60
40
20
0
(-) PVAT
+ 30 µM XE991
(+) PVAT
+ 30 µM XE991

Figure S2
